# Effect of FibroScan test in antiviral therapy for HBV-infected patients with ALT <2 upper limit of normal

**DOI:** 10.1515/biol-2020-0044

**Published:** 2020-06-22

**Authors:** Xian-Zhi Han, Shu-Feng Zhang, Jia-Yin Yi, Bin Wang, Hui-Qing Sun

**Affiliations:** Department of Gastroenterology, The Fifth Affiliated Hospital of Zhengzhou University, Zhengzhou 450052, China

**Keywords:** FibroScan, HBV infection, ALT <2 ULN, antiviral therapy opportunity

## Abstract

**Objective:**

The objective of this study is to detect the liver stiffness of hepatitis B virus (HBV)-infected patients with an alanine aminotransferase (ALT) level of <2 upper limit of normal (2ULN) by FibroScan and compare histological changes to assess the progression of liver lesions and its test results.

**Methods:**

There were 36 patients who had a liver FibroScan degree of >7.3 KD (F1), and a liver biopsy was conducted. Along with serology of liver fibrosis, indexes and hierarchical processing were used for evaluation. The correlation between these factors was analyzed.

**Results:**

The histopathological results of the liver were closely correlated with liver hardness. In the pathological diagnosis of chronic hepatitis, G represents the grade of inflammation and S represents the stage of hepatic fibrosis. Pathological examination results of H&E staining of liver tissue sections revealed that the area under the work characteristic curve of the subjects in G2S1, G2S2, G3S2, and G3S3 stages was 0.923, 0.916, 0.955, and 0.971, respectively, with diagnostic cut-off values of 9.03, 9.85, 15.14, and 30.67, respectively. Furthermore, hydroxyapatite, type III procollagen, laminin, and type IV collagen of serum fibrosis indexes are associated with liver stiffness values (*P* < 0.05).

**Conclusion:**

FibroScan can be used as an alternative to liver biopsy. It is meaningful in determining whether HBV infected patients with an ALT level of <2 ULN should receive antiviral therapy.

## 1 Introduction

The incidence of hepatitis B virus (HBV) in the young- and middle-aged population remains relatively high in China and is also the main cause of cirrhosis in China. Determining the correct timing for the administration of antiviral therapy for HBV infection plays an important role in its treatment and prognosis [1]. According to the Guidelines for the Prevention and Treatment of Chronic Hepatitis B (2015 Edition), the time of antiviral treatment for chronic HBV infection is when alanine aminotransferase (ALT) levels are >2 upper limit of normal (ULN). When the ALT level is <2 ULN, only patients with histopathological changes need to receive the antiviral treatment [2]. Liver biopsy is the gold standard for histopathological results. However, it is an invasive procedure, and as such, patient acceptance is low. Due to this, liver biopsies are unable to be routinely conducted. Furthermore, the uneven distribution of hepatic fibrosis and differences in subjective observations can often lead to misdiagnosis. An alternative to this is FibroScan, which is a non-invasive device that can be repeatedly carried out [3,4]. In patients with normal liver function, some may have occult hepatitis, hepatic fibrosis, and F2 or above and require a selective liver biopsy to determine whether antiviral therapy is necessary. Hence, FibroScan is a great alternative to these patients.

## 2 Data and methods

### 2.1 Clinical data

From July 1, 2015, to June 30, 2017, 36 patients with HBV infection who were diagnosed with hepatic fibrosis with a degree of >7.3 KD (F1 or above) underwent liver biopsy. There were 28 males and 8 females. The average age of these patients was 50.2 ± 11.3 years, with a mean body mass index of 22.53 ± 2.67. The diagnosis conformed to the diagnostic criteria within the Guidelines for the Prevention and Control of Chronic Hepatitis B (2010 Edition), which was jointly established by the Chinese Medical Association Liver Disease Branch and the Association of Infectious Diseases of the Chinese Medical Association [5]. The patients were divided into three groups, according to their liver hardness values: F1–F2 (7.3–9.7), F2–F3 (9.7–12.4), and F3 (12.4 or above).


**Informed consent:** Informed consent has been obtained from all individuals included in this study.
**Ethical approval:** The research related to human use has been complied with all the relevant national regulations, institutional policies and in accordance with the tenets of the Helsinki Declaration, and has been approved by the authors' institutional review board or equivalent committee.

### 2.2 Methods

Enzyme-linked immunosorbent assay was used to measure hepatitis B. The HBV DNA was detected by polymerase chain reaction, and patients with high values subsequently underwent a high-sensitivity test. Liver fibrosis was detected by the FibroScan502 system (France). Color Doppler ultrasound, α-fetoprotein detection, and routine biochemical tests (ALT, aspartate transaminase, total bilirubin, hydroxyapatite [HA], laminin [LN], etc.) were also conducted. Liver hardness was measured using the FibroScan system. For each patient, two measurements were taken on the same day by the same technician, once in the morning and once in the evening 2 h after dinner. The final result was the mean value of these two tests. Patients with a FibroScan result of >7.3 KD (F1) and abnormal hepatic fibrosis indexes were stratified. Patients in each group underwent a liver biopsy within 1 week.

### 2.3 Statistical analysis

The experimental data were subjected to statistical and correlation analyses using the SPSS 25.0 software. Measurement data were expressed as mean ± standard deviation (*x* ± SD) and compared among groups using univariate analysis of variance (ANOVA). Data were compared between two groups using independent sample *t*-test. The diagnostic accuracy of FibroScan results was evaluated through the receiver operating characteristic (ROC) curve. The sensitivity and specificity of the FibroScan diagnostic technique were calculated with a *P* < 0.05 that was considered to be statistically significant.

## 3 Results

### 3.1 General data of the included patients

**Figure 1:**
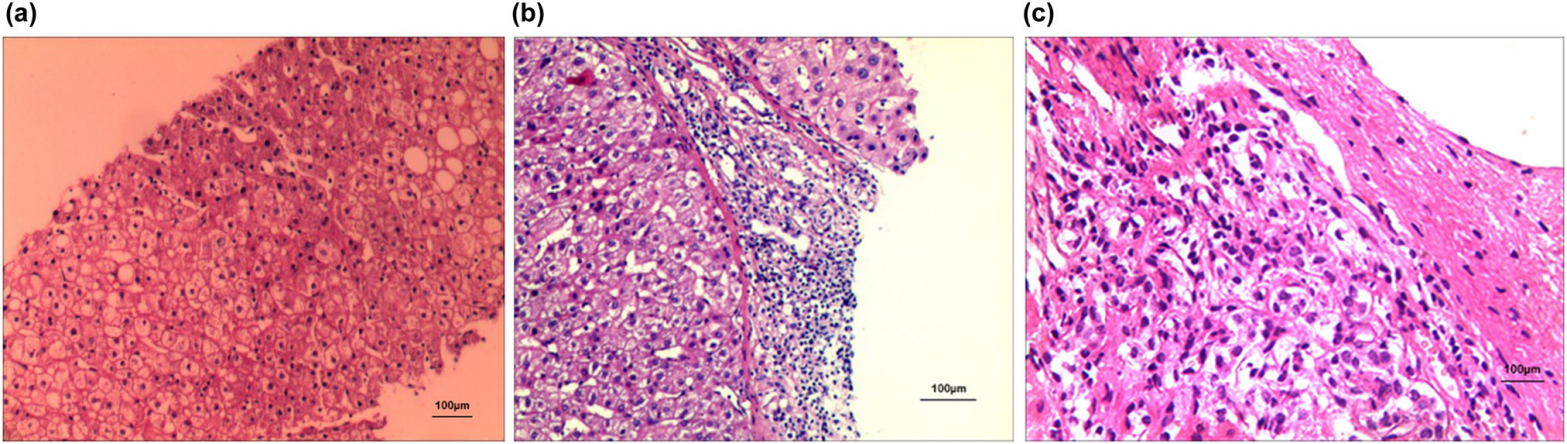
Comparison of pathological sections at three different grades: (a) G2S1 mild chronic hepatitis, (b) G2S3 mild-to-moderate hepatitis, and (c) G3S3 nodular cirrhosis.

A total of 623 chronic HBV-infected patients had an ALT level of <2 ULN. Furthermore, 66 patients had abnormal FibroScan and serological results (10.59%), and 36 patients met the inclusion criteria and underwent a liver biopsy. Among these 36 patients, 15 patients were at F1–F2 stage, 17 patients were at F2–F3 stage, and 4 patients were at F3 or above stage (Table 1). A comparison of histology sections of these three different pathological stages is presented in detail in Figure 1.

**Table 1:** Correlation between FibroScan and histopathological changes for 36 HBV-infected patients with ALT <2 ULN

Groups/pathology	*N*	G1S0	G2S1	G2S2	G3S2	>G3S3
F1–F2	15	11	2	2	0	0
F2–F3	17	2	10	4	1	0
>F3	4	0	0	2	1	1

### 3.2 Evaluation results of FibroScan diagnostic test

With the results of the 36 patients serving as the standard, the diagnostic value of FibroScan was evaluated using the diagnostic ROC curve, and the cut-off value was obtained through the Jordan index. These results suggest that the area under the curve (AUC) was >0.9 at all stages, and both the sensitivity and specificity were high. This shows that the diagnostic value of FibroScan is high (Table 2).

**Table 2:** Diagnostic indexes of FibroScan

Stages	AUC	95% confidence interval	Critical value	Sensitivity (%)	Specificity (%)
≥G2S1	0.923	0.832–1.000	9.03	91.3	84.6
≥G2S2	0.916	0.821–1.000	9.85	90.9	84.0
≥G3S2	0.955	0.866–1.000	15.14	80.0	99.8
≥G3S3	0.971	0.916–1.000	30.67	99.9	78.6

### 3.3 Correlations between FibroScan and serological indicators

The one-way ANOVA of the correlations between serological indexes and liver hardness in the 36 patients showed statistically significant (*P* < 0.05) differences in HA, type III procollagen (PCIII), LN, and type IV collagen (CIV) levels among patients with different liver hardness (Table 3).

**Table 3:** Correlation between FibroScan and serum fibrosis for 36 HBV-infected patients with ALT <2 ULN

Groups	*N*	HA (µg/L)	PCIII (µg/L)	LN (ng/mL)	CIV (ng/mL)
F1–F2	15	241.80 ± 131.70	209.36 ± 46.25	185.17 ± 18.69	210.27 ± 141.97^*^
F2–F3	17	402.36 ± 134.82^*^	225.64 ± 58.75^*^	209.48 ± 55.35^*^	260.20 ± 97.80^*^
>F3	4	483.55 ± 142.24^*▲^	267.61 ± 48.40^*▲^	251.90 ± 35.90^*▲^	288.74 ± 87.86^*▲^
*F*		9.428	7.241	5.806	7.106
*P*		0.001	0.019	0.034	0.022

Note: Compared with F1–F2, ^*^ *P* < 0.05; compared with F2–F3, ^▲^ *P* < 0.05.

### 3.4 Correlation between the FibroScan result and HBV DNA load

The high-sensitivity test revealed low virus replication in all the 36 patients. Furthermore, there was no significant correlation between liver fibrosis and virus DNA levels (Table 4).

**Table 4:** Correlation between FibroScan and HBVDNA for 36 HBV-infected patients with ALT <2 ULN

Groups	N/HBV DNA (IU/mL)	<5 × 10^2^	5 × 10^2^–10^4^	10^4^–10^6^	>2 × 10^6^
F1–F2	15	1(367)	5	5	4
F2–F3	17	1(450)	7	6	3
>F3	4	1(201)	2	1	0

## 4 Discussion

Due to hepatitis B immunization, the growth rate of HBV infection in China has decreased to a certain extent. However, the total number of infected patients remains large [6]. The early screening of hepatitis B and timely administration of antiviral therapy are important measures to reduce the risk of liver failure and death [7]. This is particularly important in patients with chronic HBV infection. According to the Guidelines for the Prevention and Treatment of Chronic Hepatitis B (2015 Edition), for patients with HBV infection, when the ALT level is <2 ULN, only those with pathological changes are required to undergo antiviral treatment [2]. Recently updated guidelines by the American Association for the Study of Liver Diseases (AASLD) recommend that patients with HBV infection and cirrhosis undergo antiviral therapy. In addition, patients with HBV DNA levels of >2,000 IU/mL regardless of ALT level should receive antiviral therapy. When the ALT level is <2 ULN, the pathological changes of the liver are taken into account to determine whether antiviral treatment is required [8,9].

Hepatic fibrosis refers to the condition when excessive extracellular matrix (ECM) deposition occurs in the liver, and the abnormal hyperplasia of a large number of fibrous tissues occurs in the portal area. This is a reversible pathological condition during the process where various chronic liver diseases develop to cirrhosis [10,11,12]. The ECM production is the core link of hepatic fibrosis. This occurs by the synthesis and decomposition of collagens in the liver, including HA, CIV, PC III, and LN, and lose dynamic balance, which causes oxidative stress in the liver to be activated and the excessive deposition of ECM [13,14]. In the current study, with the increase in hepatic hardness, HA, CIV, PCIII, and LN levels were significantly increased in the 36 patients. Previous studies have shown that the changes in HA, CIV, PCIII, and LN levels could closely reflect the activity level of hepatic fibrosis [10,15]. At present, liver biopsy remains the “gold standard” for hepatic fibrosis diagnosis, which is invasive and carries certain risks, resulting in low patient acceptance [16]. Clinically, some detection methods have a relatively high acceptance rate, which includes Doppler ultrasound, two-dimensional ultrasound, and various biochemical methods. However, the sensitivity and specificity vary. As a non-invasive detection method, FibroScan can be used as an alternative for the pathological biopsy of the liver, which is particularly useful in patients with cirrhosis [17,18].

FibroScan can assess the degree of hepatic fibrosis by measuring the transmission velocity of shear wave in the liver and evaluate the liver hardness value. This approach has a number of advantages that include being rapid, non-invasive, easy to operate, and with high safety. Furthermore, the range of the hepatic hardness value is within 2.8–75.0 kPa. FibroScan measurements are also able to monitor the dynamic changes of hepatic fibrosis at each stage and dynamically evaluate hepatic conditions in patients [19,20]. However, there are limitations, the measurement of results is sometimes affected by ascites, obesity, inflammation, cholestasis, hepatic congestion, and the size of the probe [21,22].

Antiviral treatment in patients with HBV infection has been shown to reduce the viral load [1,23], and monitoring is needed during the process of antiviral treatment [24]. A study reported that hepatic stiffness was measured to monitor the outcomes of hepatic fibrosis in antiviral therapy, and the result confirmed that long-term viral inhibition was correlated with the outcomes of hepatic fibrosis [25]. Therefore, monitoring the degree of hepatic fibrosis is useful during antiviral treatment. In China, the Guidelines for the Prevention, Management, and Treatment of Chronic HBV Infection (2015 Edition) also emphasize that FibroScan can be used to monitor hepatic hardness in chronic HBV infection, to improve the success rate and speed of detection [2]. Thus, identifying the degree of hepatic fibrosis is not only the key to knowing when to start antiviral treatment but also of importance for the evaluation during antiviral therapy and post-treatment. In our study, the accuracy of FibroScan to assess hepatic hardness in hepatic fibrosis was evaluated.

The levels of HA, LN, and serum albumin, hepatic hardness, and biopsy results were compared among all selected patients. The results revealed that relevant serum biochemical parameters and hepatic hardness were correlated with the stage and grade of hepatic biopsy (*P* < 0.005). This demonstrates the detection accuracy of FibroScan and reveals that hepatic stiffness is significantly correlated with hepatic fibrosis. However, there are limitations to the current study. This includes that all the selected patients were patients with viral hepatitis. We did not investigate whether there was a difference in hepatic hardness in patients with different types of chronic liver diseases, and further investigations are required to elucidate this.

## References

[1] Yuen MF, Chen DS, Dusheiko GM, Janssen HLA, Lau DTY, Locarnini SA, et al. Hepatitis B virus infection. Nat Rev Dis Primers. 2018;4:18035. 10.1038/nrdp.2018.35 29877316

[2] Wang GQ, Wang FS, Cheng J, Ren H, Zhuang H, Sun J, et al. The guideline of prevention and treatment for chronic hepatitis B: a 2015 update. Chinese J Liver Dis (Electronic Version). 2015;3:1–18, (in Chinese).

[3] Nakamura Y, Aikata H, Fukuhara T, Honda F, Morio K, Morio R, et al. Liver fibrosis assessment by FibroScan compared with pathological findings of liver resection specimen in hepatitis C infection. Hepatol Res. 2017 Jul;47(8):767–72. 10.1111/hepr.12815 27591427

[4] Dong H, Xu C, Zhou W, Liao Y, Cao J, Li Z, et al. The combination of 5 serum markers compared to FibroScan to predict significant liver fibrosis in patients with chronic hepatitis B virus. Clin Chim Acta. 2018;483:145–50. 10.1016/j.cca.2018.04.036 29709450

[5] Jia JD, Li LJ. The guideline of prevention and treatment for chronic hepatitis B (2010 version). J Clin Hepatol. 2011;1:113–28, (in Chinese). 21569677

[6] Meng J. An epidemiological survey on hepatitis B virus infection based on inpatients: a cross-sectional study. [D]Jilin: Jilin University; 2016.

[7] Koffas A, Dolman GE, Kennedy PT. Hepatitis B virus reactivation in patients treated with immunosuppressive drugs: a practical guide for clinicians. Clin Med. 2018;18:212–8. 10.7861/clinmedicine.18-3-212 PMC6334086 29858430

[8] Terrault NA, Bzowej NH, Chang KM, Hwang JP, Jonas MM, Murad MH. American Association for the Study of Liver Diseases. AASLD Guidelines for Treatment of Chronic Hepatitis B. Hepatology. 2016;63:261–83. 10.1002/hep.28156 PMC5987259 26566064

[9] Goyal R, Mallick SR, Mahanta M, Kedia S, Shalimar, Dhingra R, et al. Fibroscan can avoid liver biopsy in Indian patients with chronic hepatitis B. J Gastroenterol Hepatol. 2013;28:1738–45. 10.1111/jgh.12318 23808910

[10] Xiong WN, Nie J, Sun RJ, Dong ED. Organ fibrosis:an important pathophysiological process. Bull Natl Nat Sci Found China. 2015;3:172–7, (in Chinese).

[11] Chen Y, Luo Y, Huang W, Hu D, Zheng RQ, Cong SZ, et al. Machine-learning-based classification of real-time tissue elastography for hepatic fibrosis in patients with chronic hepatitis B. Comput Biol Med. 2017;89:18–23. 10.1016/j.compbiomed.2017.07.012 28779596

[12] Peeters G, Debbaut C, Friebel A, Cornillie P, De Vos WH, Favere K, et al. Quantitative analysis of hepatic macro- and microvascular alterations during cirrhogenesis in the rat. J Anat. 2018;232:485–96. 10.1111/joa.12760 PMC5807949 29205328

[13] Zhang XY, Fu YQ. Research Advances of Influential Factors on Liver Fibrosis. Med Recapitul. 2018;6:1062–6, (in Chinese).

[14] Lotowska JM, Sobaniec-Lotowska ME, Lebensztejn DM. Ultrastructural characteristics of the respective forms of hepatic stellate cells in chronic hepatitis B as an example of high fibroblastic cell plasticity. The first assessment in children. Adv Med Sci. 2018;63:127–33. 10.1016/j.advms.2017.09.002 29120853

[15] Xiong Z, Mei YF, Wang CY, Wei BZ, Liu P, Shen LH, et al. Analysis of diagnostic value of hyaluronic acid, procollagen III, procollagen IV, laminin for liver cirrhosis. Journal Of China Prescription Drug. 2018;1:137–8, (in Chinese).

[16] Schwabl P, Bota S, Salzl P, Mandorfer M, Payer BA, Ferlitsch A, et al. New reliability criteria for transient elastography increase the number of accurate measurements for screening of cirrhosis and portal hypertension. Liver Int. 2015;35:381–90. 10.1111/liv.12623 24953516

[17] Li Q, Chen L, Zhou Y. Changes of FibroScan, APRI, and FIB-4 in chronic hepatitis B patients with significant liver histological changes receiving 3-year entecavir therapy. Clin Exp Med. 2018;18:273–82. 10.1007/s10238-018-0486-5 29350286

[18] Meng F, Zheng Y, Zhang Q, Mu X, Xu X, Zhang H, et al. Noninvasive evaluation of liver fibrosis using real-time tissue elastography and transient elastography (FibroScan). J Ultrasound Med. 2015;34:403–10. 10.7863/ultra.34.3.403 25715361

[19] Viganò M, Massironi S, Lampertico P, Iavarone M, Paggi S, Pozzi R, et al. Transient elastography assessment of the liver stiffness dynamics during acute hepatitis B. Eur J Gastroenterol Hepatol. 2010;22:180–4. 10.1097/MEG.0b013e328332d2fa 19855283

[20] Wilder J, Patel K. The clinical utility of FibroScan((R)) as a noninvasive diagnostic test for liver disease. Med Devices. 2014;7:107–14. 10.2147/MDER.S46943 PMC4014361 24833926

[21] Cao JB, Chen YP, Cheng J, Deng CL, Duan XF, Gong GZ, et al. Expert consensus on clinical application of transient elastography (TE) (2015). Chinese J Liver Dis (Electronic Version). 2015;2:12–8, (in Chinese).

[22] Zheng RQ, Jin JY. Application progress of ultrasonic shear wave elastography in the hepatic disease. Organ Transplant. 2017;4:260–6.

[23] Sriprayoon T, Mahidol C, Ungtrakul T, Chun-On P, Soonklang K, Pongpun W, et al. Efficacy and safety of entecavir versus tenofovir treatment in chronic hepatitis B patients: a randomized controlled trial. Hepatol Res. 2017;47:E161–8. 10.1111/hepr.12743 27176630

[24] Chon YE, Park JY, Myoung SM, Jung KS, Kim BK, Kim SU, et al. Improvement of liver fibrosis after long-term antiviral therapy assessed by fibroscan in chronic hepatitis B patients with advanced fibrosis. Am J Gastroenterol. 2017;112:882–91. 10.1038/ajg.2017.93 28374814

[25] Wu SD, Liu LL, Cheng JL, Liu Y, Cheng LS, Wang SQ, et al. Longitudinal monitoring of liver fibrosis status by transient elastography in chronic hepatitis B patients during long-term entecavir treatment. Clin Exp Med. 2018;18:433–43. 10.1007/s10238-018-0501-x 29696462

